# Epidemiology is about disease in populations

**DOI:** 10.1007/s10654-020-00701-9

**Published:** 2020-11-28

**Authors:** Anders Ahlbom

**Affiliations:** 1 Institute of Environmental Medicine, Karolinska Institutet, Box 210, 171 77 Stockholm, Sweden; 2 Center for Work and Environmental Health, Stockholm Region, Stockholm, Sweden

Dekkers and Mulder argue convincingly that it is not possible to predict with certainty whether a given person will develop a disease or not during a specified period of time [1]. This may serve as a reminder that epidemiology is about disease in populations, rather than in individuals or patients. This editorial has to credos: First, the intrinsic core of epidemiology is that it relates cases of disease to a source population observed during a period of time, and second, epidemiology entails both subject matter knowledge and methods to generate such knowledge.

Basic descriptive epidemiological measures are taught at the beginning of every introductory course in epidemiology. These measures appear trivial on the screen in the classroom and can be taught by any teacher in 15 minutes, but it may take longer to learn them and mismatches between numerator and denominator appear again and again. This has of course been discussed extensively with respect to case-control studies but is a frequent issue also in other contexts.

The population connection was rather obvious in the early days when textbooks were organized in chapters addressing time, place, and person and when the first schools of public health were opened, and so even in John Snow’s and James Lind’s days [2]. However, when establishment of disease causation started to become a more explicit study aim, and in particular when long latency periods and multiple causes required new study designs to be developed, the population dimension of epidemiological studies became less apparent. Indeed, it is customary to report study results in terms of only relative risks with no mentioning of the basic rates and, e.g., the case-control study does not even provide rates. Yet, although not immediately visible, the population dimension remains in study designs such as the case-control study, family based designs, and other designs where the aim is to draw conclusions based on disease occurrence in populations [3].

The advent of the Covid-19 pandemic has not only promoted epidemiology and incorporated the word in everyday language, it has also provided numerous examples of the difficulties that may be attached to the seemingly trivial concept of identifying cases of disease and an underlying population properly. The purpose of this text is not to criticize individual studies but to illustrate problems, so reference to specific studies are not given: Any study comparing Covid-19 incidence across populations, e.g., defined by socio-economic status, must consider that differences in testing frequency will affect results. Likewise, if one plans to use a database of verified Covid-19 cases to look at risk factors for severe disease or death, it is essential to consider the likelihood that infected people with putative risk factors are more likely to be tested and included in the database. An example from outside the Covid-19 area is a study comparing the risk of a certain injury across levels of comorbidity. The study divided the injury cases properly according level of comorbidity but omitted to do so with the denominator and instead used the full population as denominator in all comorbidity classes. Consider also the question of how much incidence rates for myocardial infarction differ depending on number of preceding myocardial infarctions. In many settings it would be rather straightforward to divide the heart attacks by the number of preceding myocardial infarctions, but considerably more difficult to do the same thing for the denominator, the population, because the numerator and the denominator are likely to be obtained from data sources with different type of data and level of detail. The person time at risk would also have to consider whether a new event shortly after a previous myocardial infarction is a new myocardial infarction or a consequence of the preceding one.

There are many reasonable ways to describe what epidemiology is. In teaching and in textbooks something along the lines: “Epidemiology is the study of the distribution and determinants of disease in man” has been used for a long time and is still common [2]. This means that both subject matter knowledge as well as methods to generate that knowledge are entailed. It seems reasonable to use the term clinical epidemiology to the situation in which the population consists of patients and the outcome is the progress of the disease. Since epidemiologists often are called to explain what they do this is probably not entirely clear, though. It is not uncommon for clinicians and also for statisticians to omit the subject matter part of epidemiology and consider it purely a methodology, perhaps akin to, or a subset of, biostatistics. It is certainly true that epidemiology relies on biostatistics and that this reliance increases with more complex data and analytical methods. But epidemiology also has its own theory and methods and uses statistical methods in a way that to a great extent is adjusted to epidemiology. And in addition, epidemiology has the subject matter part. Most epidemiology courses are in fact courses in methods for epidemiologic research, even when called, e.g., cardiovascular epidemiology, which might explain the confusion.

Epidemiological studies are conducted for a variety of reasons. One common goal, but certainly not the only one, is to contribute to the understanding of to what extent some exposure, in a broad sense, increases the risk of a disease; thus, the goal is to learn about causation. This is achieved by comparison of disease rates in cleverly chosen populations conditioning on relevant factors. Note that the core of epidemiology, the connection between cases of disease and the source population is as important as in descriptive epidemiology. Another note is that both proficiency in epidemiologic research methods and the knowledgebase related to the specific causal question are required for success. Very likely also scientists from other disciplines are addressing related etiologic questions simultaneously from an entirely different starting point such as molecular genetics. A way to look at this is that epidemiologic research provides input to the causal assessment along with research from various other areas.

From having been a rather exclusive research field mainly exercised in departments of epidemiology, schools of public health, and in some selected research teams epidemiology has spread widely and most departments in university hospitals now have some epidemiological research directed towards their own specialty in conjunction with other research. This is a logical development because this is where some of the questions arise and where some of the needed subject matter knowledge resides, and in a sense, this infiltration is a tribute to epidemiology. Certain areas such as cancer epidemiology or air pollution epidemiology involve scientists with full command of both epidemiological research methods and the subject matter and there are chairs with corresponding titles and there is also close collaboration between expertise in different areas. Of course, there are also solitary stars doing excellent work in environments largely busy with other types of research. The downside of this successful infiltration by epidemiology is of course that it not always comes with appropriate skills, and, indeed, it may not even be recognized as epidemiology by those who conduct the work.

The advent of the Covid-19 pandemic has brought epidemiology to the attention of many, and more people than ever have a decent understanding of what it is. Indeed, quite a number of the most burning research questions stemming from the pandemic are related to when, where, and who. As has happened with other novel research topics, once the basic questions have been addressed, causally oriented questions are on wait, such as why do some patients develop severe disease but not others, questions that again require proper epidemiological research methods in conjunction with immunology, respiratory medicine, and others.

A concluding remark of this editorial would be to echo Pearce and others in their recent paper that there is a need for epidemiology to go back to its roots maintaining the link to the population that gave rise to the cases of disease regardless how defined, and linked to that, to upgrade descriptive studies [4].

Finally, back to Dekkers and Mulder [1]. They deprive us of the hope that even as successful research by epidemiologists and others takes us closer and closer to the point where ultimate prediction will be possible, there will always be a bit left, for good or bad.
